# Understanding the Mechanisms of Action and Effects of Drugs of Abuse

**DOI:** 10.3390/molecules28134969

**Published:** 2023-06-24

**Authors:** Daniela-Mădălina Ciucă Anghel, Gabriela Viorela Nițescu, Andreea-Taisia Tiron, Claudia Maria Guțu, Daniela Luiza Baconi

**Affiliations:** 1 Department of Toxicology, Carol Davila University of Medicine and Pharmacy, 20021 Bucharest, Romania; claudia.gutu@umfcd.ro (C.M.G.); daniela.baconi@umfcd.ro (D.L.B.); 2 Ward ATI—Toxicology, Paediatric Clinic 2, “Grigore Alexandrescu” Emergency Clinical Hospital for Children, 011732 Bucharest, Romania; 3 Department of Medical Semiology, Sf. Ioan Emergency Clinical Hospital, Carol Davila University of Medicine and Pharmacy, 20021 Bucharest, Romania; andreea.tiron@umfcd.ro

**Keywords:** alcohol, amphetamines, benzodiazepines, cannabis, cardiovascular toxicity, genetic susceptibility, hallucinogens, inhalants, NPS, opioids

## Abstract

Aim. Drug abuse and addiction are major public health concerns, with millions of people worldwide affected by the negative consequences of drug use. To better understand this complex issue, a review was conducted to examine the mechanisms of action and effects of drugs of abuse, including their acute and chronic effects, the symptoms of abstinence syndrome, as well as their cardiovascular impacts. Methods. The analyzed data were obtained after surveying an electronic database, namely PubMed, with no time limit, grey literature sources, and reference lists of relevant articles. Results. The review highlights the different categories of drugs of abuse, such as opioids, stimulants, depressants, hallucinogens, and cannabis, and discusses the specific ways that each drug affects the brain and body. Additionally, the review explores the short-term and long-term effects of drug abuse on the body and mind, including changes in brain structure and function, physical health problems, and mental health issues, such as depression and anxiety. In addition, the review explores the effects of drug abuse on cardiovascular health, focusing on electrocardiogram changes. Moreover, the analysis of relevant literature also highlighted possible genetic susceptibility in various addictions. Furthermore, the review delves into the withdrawal symptoms that occur when someone stops using drugs of abuse after a period of chronic use. Conclusion. Overall, this review provides a comprehensive overview of the current state of knowledge on drug abuse and addiction. The findings of this review can inform the development of evidence-based prevention and intervention strategies to address this critical public health issue.

## 1. Introduction

Recent revisions to the International Classification of Diseases (ICD)—11 have led to a reorganization of the diagnostic criteria for drug addiction or substance use disorder (SUD) [1,2]. The key characteristic of SUD is the inability to control drug use despite the adverse effects it brings about [3].

The issue of drug abuse and addiction has escalated into a significant public health concern, with an alarming increase in the number of users affected worldwide. In Europe, cannabis is the most commonly used illicit drug, followed by stimulants, such as cocaine, MDMA (3,4-Methylenedioxymethamphetamine, Ecstasy), and amphetamines, as reported by EMMCDA (European Monitoring Centre for Drugs and Drug Addiction). While the prevalence of opioid use is low, it poses significant harm through the transmission of infectious diseases, primarily among intravenous drug users [4].

In the past few decades, in the pursuit of combating drug addiction, research efforts have primarily focused on understanding the toxic mechanisms of addictive substances. A significant challenge faced by specialists is the fact that many drug users combine multiple substances, leading to non-specific symptoms. This makes it difficult to accurately identify the specific substance causing intoxication based solely on clinical examination. To gain a better understanding of how different types of drugs affect the body, it is essential to examine the crucial neurotransmitters involved, the actions of various drugs on these neuro-transmitters, and possible genetic associations. Furthermore, meta-analysis studies have demonstrated noteworthy links between serious mental illnesses, such as non-affective psychosis, major depression, and comorbidities, with drug abuse in general [5]. Notably, drug consumption has a global impact, extending beyond the general population to include truck drivers, thereby increasing the risk of accidents caused by drug abuse [6,7]. Prospective memory impairment may also be associated with recreational drug use, but studies have not been able to establish a clear link among non-intoxicated drug users [8].

This review aimed to (1) evaluate the mechanism of action for various groups of substances of abuse, (2) underline the acute and chronic side effects of the analyzed drugs, and (3) provide a future perspective on the drug abuse approach.

## 2. Results

### 2.1. Targeted Neurotransmitters

Regarding the most important neurotransmitters, details on adrenergic, dopaminergic, glutamatergic, serotoninergic, and opioidergic systems will be highlighted as follows.

Widely distributed, adrenoceptors are activated by epinephrine and norepinephrine and include three subfamilies: alpha1-, alpha2-, and beta-adrenoceptor subfamilies (beta-1, beta-2, and beta-3). The adrenoceptors mediate the actions of the sympathetic nervous system, being mostly involved in cardiac function, blood pressure homeostasis, and respiratory and metabolic processes [9,10]. With a major role in regulating cardiac contractility and heart rate, β*1-adrenoceptors* are mostly distributed in the myocardium (leading to an increase of heart rate, increase in contractility, conduction, velocity, and automaticity), kidneys (leading to renin secretion), adipose tissue (leading to lipolysis), posterior pituitary (antidiuretic hormone secretion), salivary glands (amylase release), and sympathetic nerve terminal (increase neurotransmitter release) [9,10,11]. β*2-adrenoceptors* mediate vascular smooth muscle dilation leading to the relaxation of vascular, uterus, bladder, lung, and gastrointestinal smooth muscles. On skeletal muscle, the activation of β2-adrenoceptors leads to increased contractility, glycogenolysis, and K^+^uptake. In addition, β2-effects are insulin secretion (pancreas), relaxation of the splenic capsule, amylase secretion (salivary glands), and increased secretion (lungs) [9,10]. Almost similar to β2-receptors, the β*3-adrenoceptors* are distributed in adipose tissue (leading to lipolysis), smooth muscles (producing relaxation), cardiac tissue (heart failure and hypertension), the central nervous system (leading to psychiatric disorders), and skeletal muscle (producing thermogenesis) [10]. Given their global expression in the cardiovascular, respiratory, and central nervous systems, the side effects induced by the activation of the adrenoceptors include cardiac disorders, trembling, nervous tension, headaches, and muscle cramps [10,11,12]. α*-adrenoceptors* are widely distributed, involved in cardiovascular function and modulating sympathetic activity (central and peripheral nervous systems). The activation of α*1-adrenoceptors* leads to contraction (on smooth muscle), glycogenolysis, gluconeogenesis, and ureagenesis (on the liver), an increased force of contraction (myocardium), increased locomotor activity and neurotransmission (central nervous system), glucogenesis (kidney), glycogenolysis (adipose tissue) [9,12]. α*2-adrenoceptors* modulate peripheral resistance. These are distributed in the sympathetic nerve terminal leading to inhibition of norepinephrine release in the central nervous system leading to issues with sedation, adipose tissue (inhibition of lipolysis), eyes (decreased intraocular pressure), kidneys (inhibition of renin release), and pancreatic islet cells (inhibition for insulin release) [9,12].

Dopamine is a monoamine catecholamine neurotransmitter and hormone with effects on emotions, cognition, movement, endocrine regulation, and the reward system [13,14]. Current theories outline the mesolimbic dopamine system as having an important role in substance use disorders. In this regard, the primary reinforcing effects of abuse substances have been linked to the elevation of dopamine in the nucleus accumbens by either stimulating the dopamine neurons in the VTA or inhibiting the reuptake of the dopamine in the NAc [3]. Dopamine-receptors (DA-receptors) are widely distributed in the brain (mainly in the central nervous system and peripheral nervous system), play a key role in reward-related phenomena (in the ventral tegmental area), and are divided into two pharmacological families: D1-like receptors (D1R subtype—with effects on memory, attention, locomotion, impulse control, and regulation of renal function, and D5R subtype—with effects on decision making, cognition, attention, and renin secretion) and D2-like receptors (D2R subtype—with effects on locomotion, learning, attention, memory, sleep, D3R subtype—with effects on cognition, sleep, impulse control, attention, and DRD4 subtype—with effects on cognition, sleep, attention, and impulse control) [13,14,15,16]. The distribution and function of peripheral dopamine receptors have been described by Missale et al. in 1988 and outlined effects such as inhibition of norepinephrine release (D2-like) and vasodilation (D1-like) on blood vessels, inhibition of aldosterone secretion (D2-like), stimulation of epinephrine/norepinephrine release (D1-like) and inhibition of epinephrine/norepinephrine release (D2-like) on the adrenal gland, increase of filtration rate (D1-like), stimulation of renin secretion (D1-like), inhibition of Na+ reabsorption (D1-like), inhibition of vasopressin action (d2-like), and inhibition of norepinephrine release (D2-like) on kidneys [14].

Including two receptor families (ionotropic glutamate receptors—iGluRs and metabotropic glutamate receptors—mGluRs), the glutamatergic transmission is controlled by glutamate dynamics, which acts as a primary excitatory neurotransmitter and as a key neuromodulator in synapses [17,18,19]. Glutamate acts on NMDA (*N*-methyl-d-aspartate), AMPA (α-amino-3-hydroxy-5-methyl-4-isoxazole propionic acid), kainite and metabotropic receptors leading to neurodegeneration and excitotoxicity [18,19].

Another widely (in the central nervous system and periphery) distributed monoamine involved in various behavioral disorders, such as major depression, mania, autism, schizophrenia, anxiety, obesity, and pain, is serotonin (5-hydroxytryptamine, 5-HT), a neurotransmitter with action on serotoninergic transmission [20]. Highly expressed in the human limbic system, the 5-HT receptors include seven families (coded 5-HT1–5-HT7) with approximately 15 subtypes. The action of serotonin reflects in sensory, autonomic, and motor systems, arterial pressure, recognition, memory, and sexual behavior [20,21].

Potential pathologies of the central nervous system have also been linked to the action of γ-aminobutyric acid (GABA), whose inhibitory action is mediated by chloride transporters. The main types of GABA receptors are GABAA (ionotropic receptor), GABAB (metabotropic receptor, including GABAB1 and GABAB2 subunits), and GABAC (ionotropic receptor) [22].

The Cannabimimetic transmission is primarily mediated by two members of the G-protein coupled receptors, the cannabinoid receptors, but several other receptors (ranging from other G-protein-coupled receptors to ion and nuclear receptors) are also involved in the interaction with the cannabinoids [23]. Encoded by the gene CNR1 and consisting of 472 amino acids in humans, the CB1 receptor is predominantly distributed in the brain and skeletal muscle. Due to the higher expression in the liver and pancreas, the main effects manifested in metabolism [23]. The second cannabinoid receptor, CB2-receptor, is encoded by the gene CNR2 (consisting of 360 amino acids) and is predominantly expressed in the testis and less in the brain [23]. Due to their wide distribution in the human body, these cannabinoid receptors are involved in various neural activities: learning and memory, neurodegeneration, addiction, epilepsy, appetite, and stroke [23].

Another system highly expressed in the reward circuitry is the opioid system, comprising endogenous ligands and multiple opioid receptor types (belonging to the G-protein coupled receptors). The main opioid receptors are µ, κ, and δ receptors. Activated by a large variety of molecules (endorphins, enkephalins, dynorphins, alkaloids, and synthetic molecules), these receptors are crucial modulators of the substance abuse disorder leading to various side effects, including tolerance and addiction [3,24].

**Figure 1:**
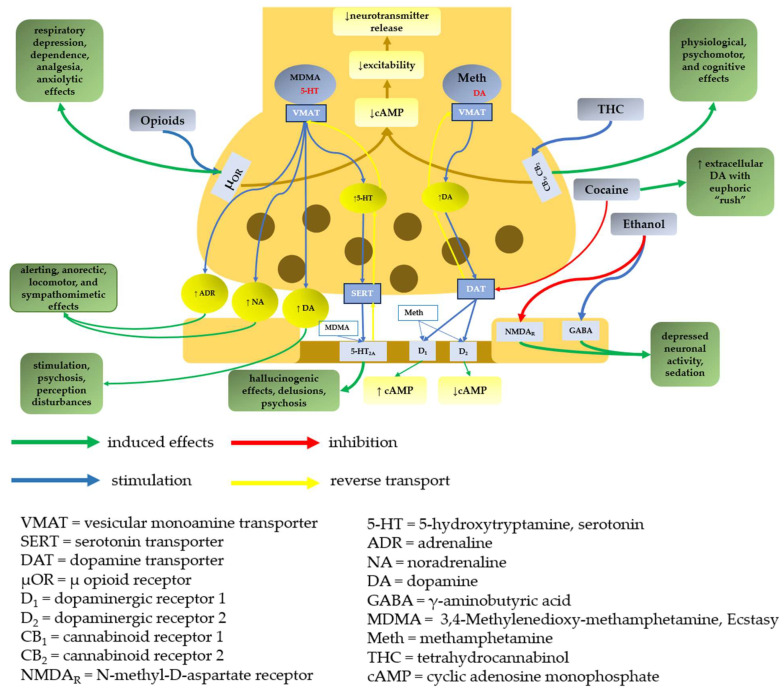
Molecular targets and primary outcomes resulting from the effects of substances of abuse on neuronal terminals.

Molecular targets and primary outcomes resulting from the effects of substances of abuse on neuronal terminals are represented in Figure 1.

### 2.2. Effects of Drugs of Abuse on the Brain

Substances of abuse have a wide range of effects on the human body, including behavioral, biochemical, and toxic organic consequences, particularly impacting the brain. In terms of behavioral effects, drug abuse can lead to altered cognitive function, impaired judgment, and changes in mood and emotions. These substances often disrupt the brain’s reward system, leading to addictive behaviors and compulsive drug-seeking. On the biochemical level, substances of abuse can interfere with the normal functioning of neurotransmitters, such as dopamine, serotonin, and gamma-aminobutyric acid (GABA), affecting mood regulation, reward processing, and overall brain function. Additionally, chronic drug abuse can result in toxic organic effects on the brain, leading to structural and functional damage.

Analyzing the included papers in this research (according to inclusion criteria, Section 4), a few data regarding the effects of drugs of abuse on the brain were extracted. By focusing on meta-analysis studies exclusively (with no time limit and ‘free-full text’), we aimed to provide an unbiased and rigorous analysis of the available evidence.

To obtain a comprehensive overview of existing literature, we also conducted an extensive search on the PubMed database. Employing the search query ‘meta-analysis’ + ‘full-text’ + wording ‘effects of drugs of abuse on the brain’, we anticipated a vast number of relevant studies. However, the search yielded a relatively limited number of results, specifically 10 studies (of which two were irrelevant topics). This unexpected outcome highlights the significance of our research, as it indicates a potential gap in the current body of literature concerning the effects of substances of abuse on the brain based on a meta-analysis.

#### 2.2.1. Behavioral Effects

##### Depressants

*Heroin and other opioids* differentiate themselves by their addictive properties. Due to increased dopamine levels, opioids use leads to repetitive drug use and, therefore, to addictive behavior. As behavioral changes, opioids also induce euphoria, sedation, and dysphoria (in the case of withdrawal syndrome) [25].

*Alcohol*, being a general central nervous system (CNS) depressant, induces dose-dependent sedation (alertness is first affected, followed by judgment) [25]. Although the users experience a stimulating phase in moderate consumption (manifested as relaxation, loss of inhibition, well-being, emotional arousal, and pleasure), in higher doses, the brain stem and cerebellum are affected, which leads to depression, aggression, mood swings, sadness, and anger [25].

##### Stimulants

*Amphetamines* lead to intense euphoric effects; therefore, it is illegally used for relaxation but also for their effect on increasing alertness and reducing fatigue. By acting on the reward pathway (as a result of synaptic dopamine increase), methamphetamine, amphetamine, and MDMA induce pleasure [25]. Effects such as psychosis and perception disturbances are mediated by dopamine, and alerting is associated with noradrenaline, while serotonin is responsible for psychosis and delusions [25]. Due to neurotoxicity, MDMA also induces aggression and mood change [25].

Used as a recreational drug for its intense euphoric effects, *cocaine* (due to its stimulant action) increases alertness but also self-confidence. The euphoric ‘rush’ is determined by the increases in dopamine levels and includes euphoria, confusion, agitation, and hallucination, while falling dopamine levels determine dysphoric ‘crash’ [25].

##### Particular Mechanism

Behavioral effects of *cannabis* consumption include alterations in perception, cognition, motor behavior, memory, and learning, but also psychotic episodes in the case of long-term use [25].

Regarding the behavioral effects, among the analyzed paper, only one meta-analysis-based study was identified, and it outlined the fact that illegal drugs (cocaine, heroin) are associated with a more severe degree of harm, dependence, and criminal behavior than legal ones (nicotine, alcohol) [26].

#### 2.2.2. Biochemical Effects

##### Depressants

*Heroin and other opioids*, by their interaction with μ-opioid receptors, lead to increased synaptic dopamine levels (due to disinhibition of dopaminergic neurons) involved in the reward mechanism [25].

*Alcohol* acutely depresses neural activity by enhancing GABAergic neurotransmission and inhibiting the excitatory *N*-methyl-d-aspartate receptors, which leads to sedation. At the reward pathway, alcohol releases the opioid neuropeptides which leads to the disinhibition of dopamine release in the nucleus accumbens. Due to neuroadaptations at the ion channel, long-term use of alcohol leads to withdrawal syndrome when alcohol is no longer present [25].

##### Stimulants

*Methamphetamine* increases synaptic levels of the monoamine neurotransmitters (noradrenaline, dopamine, and serotonin) [25]. *Amphetamine* impairs the active transport of the monoamines into the synaptic vesicles, leading to vesicular biogenic amine content depletions. It also slows down catecholamine metabolism and inhibits dopamine synthesis. [25]. *MDMA* increases the levels of dopamine and noradrenaline but also has hallucinogenic effects [25].

*Cocaine* consumption leads to increased extracellular dopamine (associated with pleasure). In addition, cocaine leads to the accumulation of noradrenaline and serotonin at postsynaptic receptors but also increases catecholamine levels in the blood [25].

##### Particular Mechanism

*Cannabis* activates cannabinoid receptors and leads to a decrease in adenylate cyclase activity, leading to decreases in electrical excitability and neurotransmitter release. It also decreases gamma-aminobutyric acid (GABA) release, which leads to an increase in synaptic dopamine levels (similar to opioids) [25].

In terms of meta-analysis studies, an accepted theory that has been modeled and applied in drug addiction is based on the reward prediction error (RPE) and targets dopamine levels [27]. Using pharmacological magnetic resonance imaging (phMRI), measurements in the striatum based on micro-dialysis showed a smaller increase in extracellular dopamine as a response to cocaine in younger rats compared to adults [28]. Differences in striatal dopaminergic levels (in nucleus accumbens and caudate putamen) were assessed in rats, and no significant sex differences in basal levels or in dopaminergic response to substances of abuse were found [29].

#### 2.2.3. Toxic Organic and Functional Effects

A meta-analysis of 64 neuroimaging studies outlined that the alterations in neural responses appear in the brain of individuals who use substances of abuse [26]. Moreover, these alterations depend on the type of drug, giving rise to specific brain activation patterns: the use of legal substances (nicotine, alcohol) leads to more frequent activity of the medial dorsal anterior cingulate cortex (dACC), while the use of illegal drugs (cocaine, heroin) leads to more frequent activity in the left posterior inferior temporal gyrus (pITG), anterior hippocampus/amygdala, in the medial calcarine cortex and precuneus, the right caudate/nucleus accumbens, and the left midbrain (VTA) [26]. In addition, the study correlated neural responses with the class of substances and the treatment status of the participants, showing that the caudate nucleus is frequently activated in the case of treatment-seeking subjects, specifically for legal substances (nicotine, alcohol), while the right thalamus is frequently activated in the case of not-treatment seeking subjects, specifically for illegal substances addicts (cocaine, heroin). Additionally, the study correlated severe addiction in the case of illegal drugs (cocaine, heroin) with frequent activation of the subcortical reward pathway [26]. Another meta-analysis outlined altered patterns of brain gray matter structure (such as the increased volumetric pattern in the left putamen), pointing to drug-related adaptions in neural systems, such as the hyperactive reward system, as the trigger for the neuropathological processing in substance use disorders (stimulants, cannabis, alcohol, tobacco, inhalants, and poly-consumption) [30]. Moreover, alterations of white matter (reduced volume and axonal integrity) have been identified in patients with alcohol use disorder, affecting key brain structures of the cingulum, corpus callosum, fornix, and internal capsule, alterations that lead to various neuropsychological deficits (motor, cognitive, affective, and perceptual functions) [31]. A meta-analysis conducted on seven studies outlined alterations in molecular imaging of serotonin transporters in ecstasy/polydrug users, namely serotonin axons with the longest projections from the raphe nuclei appeared to be most affected by drug use [32]. In addition, macrostructural brain alterations could be induced by cannabis use [33]. The neurotoxicity of cannabis use was also assessed through a meta-analysis study outlining significant grey reduction in the whole hippocampus [34]. A meta-analysis study used pharmacological magnetic resonance imaging (phMRI) to determine the responses of various substances of abuse (cocaine, methylphenidate, amphetamine, dihydrexidine, and quinpirole) to the dopamine transporter (DAT), dopamine releaser, selective D1-agonist, and D2/D3-agonist. Primarily positive cerebral blood volume (rCBV) changes in the dopaminergic circuitry were induced by cocaine or methylphenidate in adult rats, while negative rCBV changes were identified in young rats [35]. Structural changes in the gray and white matter of the brain were also linked to cannabis use, depending on the severity of dependence (young cannabis users showed a decrease in volume of the right amygdala and the hippocampus on both sides of the brain) and the amount of cannabis consumed weekly [36]. In addition, a loss of axonal integrity (reduction of fiber pathways) in the area of the right fimbria and bilaterally in a region of the corpus callosum, a major decrease in the fiber bundle from the splenium of the corpus callosum to the right precuneus were outlined in cannabis users [36]. In a meta-analysis study based on neuroimaging literature, it has been observed that certain neural components (anterior cingulate cortex, orbitofrontal cortex, dorsolateral prefrontal cortex, striatum, insula, and somatosensory cortex) related to risk assessment may not operate at their best capacity in individuals with substance use disorders (alcohol, tobacco, marijuana, cocaine, heroin, and amphetamine) (SUDs) [37]. Examining post-error slowing in addiction, a meta-analysis study revealed that post-error slowing could be a valuable and straightforward indicator of cognitive control dysfunction in individuals with substance use disorder. This measure has the potential to be easily implemented and may be associated with irregular brain activity in regions responsible for cognitive control, such as the anterior cingulate cortex [38]. Comorbid substance abuse in schizophrenia leads to a more intense emotional experience in patients. The results of the study outlined activations of the right superior parietal cortex as well as the left medial prefrontal cortex more strongly in comorbid substance abuse (nicotine, caffeine, alcohol, cannabis, and cocaine) than in single-diagnosis patients [39]. Another meta-analysis study assessed the correlation between the effects of substances of abuse (stimulants, alcohol, nicotine, opioids, and others (cannabis, inhalants, and polysubstance use)) and oxidative and antioxidative stress markers based on 61 studies, of which four studies were conducted on brain tissue. The analysis specifically associated substance use disorder (SUD) with higher levels of oxidant markers (malondialdehyde, thiobarbituric acid reactive substances, and lipid peroxidation) and lower antioxidant markers (superoxide dismutase and the total antioxidant capacity) [40]. Another study demonstrated that alcohol and other drugs of abuse (heroin, cocaine) could induce NFkB (nuclear factor kappa light chain enhancer of activated B cells) activity and cytokine expression in the brain [28].

**Figure 2:**
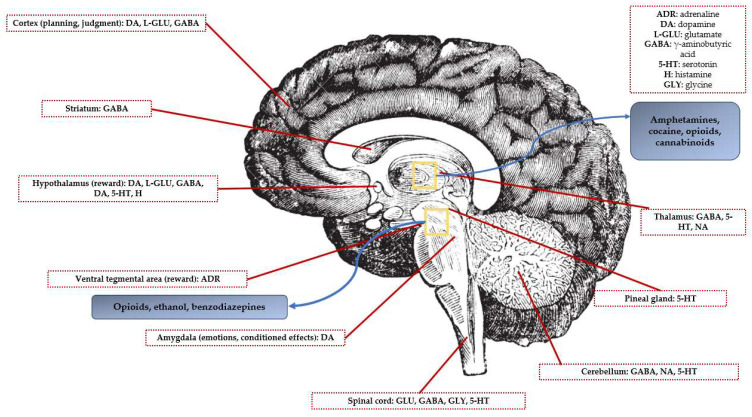
Schematic diagram of the human brain highlighting the main brain areas and neurotransmitter pathways implicated in reward processes.

An illustrative diagram showcasing key brain areas and neurotransmitter pathways involved in reward processes within the human brain is represented in Figure 2.

### 2.3. Genetic Susceptibility and Addiction

Genetic susceptibility has also been linked to addiction. Our survey on the PubMed database has revealed a few studies showing associations between drug dependence and various genes [41,42,43,44,45,46]. In this regard, a meta-analysis on genome-wide association studies (GWAS) indicated 348 genes that are linked to addiction susceptibility [41]. In the case of alcohol, a meta-analysis of 91 studies outlined a strong association between the alcohol dehydrogenase 1B gene (located on chromosome 4q21–q23 and involved in the metabolization of alcohol into acetaldehyde) and alcohol dependence [42]. Another meta-analysis has linked alcohol dependence with the gamma-aminobutyric acid A receptor a2 gene (GABRA2), explaining the inhibitor effect of alcohol on GABAergic transmission in the ventral tegmental area (VTA, heterogeneous brain structure with a central role in reward-seeking and processing, learning, motivation, and neuropsychiatric disorders [15]) of the brain [43]. In addition, another meta-analysis of genome-wide association data of 13 cohorts identified four genes significantly associated with lifetime cannabis use: NCAM1 (neural cell adhesion molecule 1, involved in the dopaminergic transmission and linked to smoking behavior, nicotine, alcohol, heroin, and other substances dependence), CADM2 (synaptic cell adhesion molecule, involved in processing speed, autism disorder, and body mass index), SCOC (a gene that encodes a short coiled-coil domain-containing protein with a role in the regulation of gene expression), and KCNT2 (potassium channel, subfamily T, member 2) (encodes a potassium voltage-gated channel, also linked to cocaine dependence) [44]. Although previously linked to substance dependence, the cannabinoid receptor 1 (CB1, with a direct role in the dopaminergic reward pathway) has been associated with cocaine addiction as well, being relevant for relapse into cocaine-seeking behavior [45]. Another meta-analysis outlined associations of the 5-hydroxytryptamine receptor (5-HTR), encoded by the HTR1B (5-HT1B) gene, with drugs of abuse, such as alcohol, cocaine, and heroin, implying that the HTR1B gene might confer susceptibility to substance use disorders [46]. A similar study associated the HTR2A gene with alcohol and heroin use. The role of the 5-HT2A receptor is related to both presynaptic (ventral tegmental area, VTA and nucleus accumbens, NAc) and postsynaptic activation [47].

In addition to genetic susceptibility, other correlations, such as the correlations between various demographic (sex, education) and psychological factors (such as personality, low self-esteem, inferiority complex, and violence) and drug abuse (in general), were proved to be statistically significant [48,49]. The outcomes of the study outlined a statistically highly significant negative correlation between the level of education and drug use (Pearson test, R (38) = −0.9282; *p* < 0.00001). In addition, males were proved to be more prone to drug consumption (Pearson test, R (38) = −0.603; *p* = 0.000038), while violence was statistically significantly correlated to drug use (Pearson test, R (38) = 0.4364; *p* = 0.004875) [48]. Alexithymia (inability to recognize and verbalize emotions) was associated with drug consumption, with alexithymic heroin addicts reporting more polysubstance abuse (opiate use, other than heroin and benzodiazepine) than non-alexithymic individuals [49].

### 2.4. Mechanisms of Action and Effects of Drugs of Abuse

Taking into account the mechanisms of action of the various classes of substances of abuse [50], in the forthcoming chapter, a comprehensive exploration will be presented, delving into the acute and chronic effects of drugs, along with an in-depth examination of the withdrawal syndrome. The discussion will focus on two widely accepted classes of drugs: depressants and stimulants. However, the particular mechanism of action needs to be addressed separately, especially for cannabis, hallucinogens, and the heterogeneous group of new psychoactive substances. This meticulous analysis will shed light on the specific physiological and psychological ramifications associated with each class, elucidating the diverse range of immediate and long-term consequences that arise from their use. By scrutinizing the intricate interplay between these drug categories and the human body, a nuanced understanding of the distinct acute and chronic effects, as well as the intricate nature of the withdrawal syndrome, will be highlighted.

#### 2.4.1. Mechanisms of Action

##### Depressants

*Heroin and other opioids* act on opioid receptors that hold high stereospecificity: Miu (µ1-3), Kappa (k1-2), and Delta (δ1-2). MORs—mu-opioid receptors are located in the cerebral cortex, thalamus, accumbens nucleus, and basolateral amygdala. The activation of the aforementioned receptors produces supraspinal and spinal analgesia, increased prolactin release (µ1), respiratory depression (µ2), decreased gastrointestinal motility (µ2), sedation, miosis, euphoria (by binding of endorphins), drug dependence (by triggering euphoria and stimulus) immunosuppression (µ3), and hyperalgesia (µ1 and µ2) [3,24,51,52,53]. By the time the addictive behavior develops, poor decision-making and impaired cognition shift from goal-oriented behaviors to common behaviors and lead to compulsive drug use [25,53]. KORs—kappa opioid receptors—are located in the hypothalamus and bind to dynorphins and trigger dysphoric and sedative effects, which can trigger anti-reward effects (producing dysphoria). KOR activation reduces dopamine release [3,25,53]. DORs—delta-opioid receptors—are located in the basal ganglia. These opioid receptors bind to encephalins and induce anxiolytic effects [3,24,25,53].

*Alcohol* is a CNS depressant that manifests its biological effects by its action on the GABAergic system (GABA), on GABA_A_ and GABA_C_ receptors, generating inhibition in the Central Nervous System (CNS), manifested by sedation, loss of inhibition, and relaxation [25]. The sedative effects of alcohol can be explained by enhancing GABAergic neurotransmission and inhibiting the excitatory *N*-methyl-d-aspartate (NMDA) receptors [25]. It also has an action on opioidergic and dopaminergic neurotransmitters. Acting on the µ receptor induces effects such as pleasure, satisfaction, and increased interest in alcohol consumption. In addition, alcohol has effects on serotonergic neurotransmitters and causes the installation of a general state of well-being (action on the 5-HT3 receptor) [54,55]. A meta-analysis study aimed to assess the dose-response effect of alcohol on blood pressure, and the results outlined that acute alcohol consumption leads to a decrease in blood pressure, an effect that usually lasts up to 12 h. The possible explanation for this effect is the increase of vasodilation induced through nitric oxide, acetaldehyde, and insulin increased production. Additionally, high doses of alcohol could lead to the reduction of 20-hydroxyeicosatetraenoic acid (vasoconstrictor that inhibits sodium reabsorption in the proximal and distal tubules of the kidney) [56].

*Benzodiazepines* act either by potentiating GABA-mediated inhibitory neurotransmission or by inhibiting adenosine reuptake in neurons. Benzodiazepines can activate the type 2 site of the GABA_A_ receptor, explaining the anxiolytic and muscle relaxant action, and the type 1 site leading to effects such as sedation, amnesia, and anticonvulsant action [22,51,57].

*Inhalants*, despite being classified as depressants, exhibit narcotic effects on the central nervous system. When these substances are inhaled, they can induce a range of psychoactive and narcotic effects due to their depressant properties. Inhalants encompass a variety of volatile substances, such as solvents, aerosols, gases, and nitrites, which can be found in common household products, industrial chemicals, and certain medical preparations. Upon inhalation, the vapors of these substances rapidly reach the lungs and enter the bloodstream, eventually reaching the brain. Inhalants have a direct impact on neurotransmitters and receptors, primarily targeting the gamma-aminobutyric acid (GABA) receptors. This interaction results in a dampening effect on neuronal activity, leading to a general slowing down of brain function [58].

##### Stimulants

Psychoactive *phenethylamines* are a large group of molecules, including amphetamines, methamphetamines, and designer amphetamines (the most popular one being MDMA—methylenedioxy-methamphetamine, also known as ‘Ecstasy’) [59]. Their mechanism is explained by the interaction with the dopaminergic, noradrenergic, and serotoninergic systems, increasing synaptic levels of dopamine, noradrenaline, and serotonin. [25,59]. *Amphetamines* are involved in the release of monoamines [60]. Amphetamine is a substrate for the dopamine transporter (DAT) (due to the similarity of structure to dopamine) and also interferes with the vesicular monoamine transporter-2 (VMAT-2) [25]. *Methamphetamine* increases synaptic levels of dopamine, serotonin (5-HT), and noradrenaline. It also has α and β-adrenergic agonist effects [25,59,61]. Although similar in structure to methamphetamine, *MDMA* has significantly fewer CNS stimulant properties than methamphetamine [25]. Studies have proved that the use of MDMA could increase sociability outcomes, such as feeling talkative, loving, and friendly, therefore acting as a social catalyst [62]. MDMA also has mild hallucinogenic properties [25]. A meta-analysis demonstrated the reduction of executive functioning in MDMA users, outlining the potential serotoninergic neurotoxicity [32]. Significant SERT (serotonin transporter) reductions in the neocortical and limbic regions were highlighted in 11 out of the 14 regions, with the greatest effects observed in the occipital cortex. MDMA affects the serotonin axons with the longest projections from the raphe nuclei [32].

Along with phenethylamines, *piperazines* induce stimulant and hallucinogenic effects due to their chemical structure that triggers the release of dopamine and norepinephrine. In addition, piperazines inhibit the uptake of monoamine.

*Synthetic cathinone* is a broad class of compounds that mimic the effects of amphetamines (they have effects on the neurotransmitters monoamine, dopamine, norepinephrine, and serotonin) [63]. These substances (e.g., cathinone, mephedrone) have psychostimulant action and induce euphoria and increased alertness in case of initial or low-dose consumption [25]. Being part of the NPS group, despite their stimulant action, synthetic cathinones are treated separately.

A stimulant drug that also needs attention is *khat* (the buds and leaves of the plant *Catha edulis*). This activates the sympathetic central nervous system through its main active constituents: cathinone and cathine (structurally and functionally similar to amphetamine) [64].

*Cocaine* acts as a blocker of the presynaptic transporters responsible for the reuptake of serotonin, noradrenaline, and particularly dopamine (associated with pleasure and movement), which causes psychoactive and sympathomimetic effects [25]. The high addictive potential of cocaine is correlated with increased dopaminergic activity along the mesocorticolimbic pathways, with predominance in the ventral tegmental area and projection to other brain locations, including the nucleus accumbens, with an important role in the rewarding and addictive properties of cocaine and other drugs. Moreover, cocaine directly targets adrenergic, glutamatergic *N*-methyl-d-aspartate (NMDA), and sigma and kappa opioid receptors [65]. The blockade of κ opioid receptors selectively attenuated increased cocaine self-administration in rats with extended access to cocaine [66]. In addition, it has recently been demonstrated that, following self-administration in rats, cocaine modulates hippocampal mu opioid receptors [67].

##### Particular Mechanism

*Cannabis* is a complex drug that can depress, excite, and impair the central nervous system, therefore having a distinctive pharmaco-toxicological profile. This contains various chemical compounds, with the most notable one being delta-9-tetrahydrocannabinol (THC). Cannabis manifests its psychoactive effects, such as euphoria and relaxation, by acting on the endocannabinoid system, specifically on the CB1 and CB2 cannabinoid receptors [68,69,70,71]. The repetitive use of cannabis can be attributed to the interaction between THC and presynaptic CB1 receptors situated on inhibitory GABAergic interneurons in the reward pathway. This interaction results in a reduction in the release of gamma-aminobutyric acid (GABA), leading to the disinhibition of dopaminergic neurons. Consequently, there is an elevation in synaptic dopamine levels, similar to the effects observed with opioid drugs of abuse [25].

*Synthetic cannabinoids* represent a class of substances of abuse that mimic the effects of cannabis and are included in the complex group of new psychoactive substances. Their mechanism of action can be explained by the action on the cannabinoid system [72]. The CB1 cannabinoid receptor [23,68,69] is located in the central and peripheral nervous system, in the bones, heart, liver, lungs, vascular endothelium, and reproductive system, and the CB2 cannabinoid receptor [23,70] is mainly localized in the immune system but also the central nervous system (but at lower levels than CB1). Synthetic cannabinoids activate CB1 receptors which lower cellular cyclic adenosine monophosphate (cAMP) and thus elicit cannabimimetic responses (due to their complete agonist action on cannabinoid receptors). They induce THC-like effects but have stronger and longer-lasting effects [73]. Synthetic cannabinoids cause agitation, irritability, confusion, hallucinations, delusions, and psychosis [74].

*Tryptamines* are tryptaminic hallucinogens (the most popular being LSD—lysergic acid diethylamine) and include monoamine alkaloids that act on 5HT2A (as an agonist) and inhibit serotonin reuptake. Although studies have demonstrated the potential therapeutic use of classic serotoninergic psychedelics in mood disorders and depressive symptoms [75], adverse effects need to be taken into account. Other hallucinogenic drugs, such as LSD, act by binding strongly to human serotonin (5-hydroxytryptamine (5-HT)) 5-HT1A, 5-HT2A, 5-HT2C, dopamine D2, and α2 adrenergic receptors and less strongly at α1 adrenergic receptors, D1, and D3 generating [76,77] arrhythmias, hypertension, heart rhythm disorders, severe hyperthermia, convulsions, visual disturbances, synesthesia, extreme mood disorders, depersonalization, disorders of perception of time and space.

Lastly, *dissociative anesthetics* induce distorted perception (of sight and sound) and dissociation by acting on 5HT2A (as an agonist) and NMDA (*N*-methyl-d-aspartate) receptor (as an antagonist) [78].

*New Psychoactive Substances* represent a complex new group of substances of abuse that, being structurally different from illicit drugs, escape legislative control. Still, their psychoactive effects mimic illicit drugs. Although definitions of NPS can vary worldwide, these include defined groups of drugs as follows: plant-based substances, synthetic cannabinoids, cathinone, hallucinogens, synthetic opioids, phenethylamines, piperazines, and synthetic benzodiazepines. Because these tend to be analogs of existing controlled drugs, NPS are known by the general population as ‘legal highs’, ‘research chemicals’, and ‘designer drugs’ [79,80]. Being a heterogenous group of substances of abuse, NPS are difficult to be classified as stimulants or depressants.

**Figure 3:**
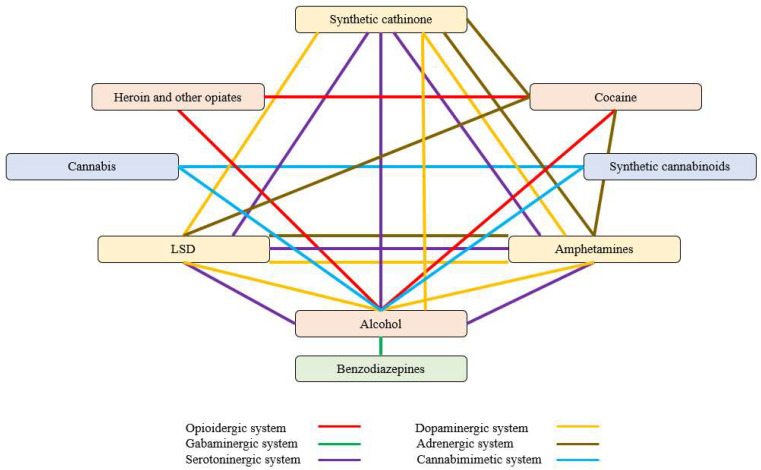
Pharmacological network structure by selectivity on receptors/systems.

Based on the above-mentioned mechanism of action for each type of analyzed drug, the selectivity of various substances of abuse on receptors/systems is synthesized in Table 1 below and represented in Figure 3.

#### 2.4.2. Effects of Drugs of Abuse

##### Acute Effects

1. Depressants

*Heroin and other opioids*. Acute intoxication with opioids manifests through a diverse array of symptoms and effects on the body. Individuals experiencing opioid intoxication may exhibit sedation, a state of drowsiness, and reduced arousal [25,53,81]. Nausea and vomiting are common symptoms that can accompany opioid use, contributing to feelings of discomfort and gastrointestinal distress [81,82]. Dizziness (a sensation of lightheadedness or imbalance) and euphoria (a state of intense happiness or well-being) can appear [25,51,53,81]. This pleasurable sensation may contribute to the addictive potential of these substances. Pruritus (or itching of the skin) and flushing (characterized by a sudden reddening or warmth of the skin) can be observed during opioid intoxication. Constipation, a common and well-known effect, results from the opioids’ ability to slow down intestinal motility [25,51,53,81,82]. Bradycardia and hypothermia may also occur as physiological responses to opioid ingestion [83]. Miosis is a classic sign of opioid intoxication and can aid in recognizing opioid overdose. However, one of the most critical and potentially life-threatening effects of opioid intoxication is respiratory depression [25,53,81]. Opioids suppress the central nervous system, including the respiratory centers in the brain, leading to slowed and shallow breathing. Severe respiratory depression can result in respiratory arrest and can be fatal if not promptly addressed [51]. Recognizing the signs and symptoms of acute opioid intoxication is crucial for timely medical intervention and overdose management. Prompt medical attention, including the administration of opioid-reversing agents, such as naloxone, is vital in cases of opioid overdose to restore normal respiration and prevent fatality [25].

*Alcohol*. When individuals consume alcohol in excessive amounts, they may experience a range of symptoms and complications: mild visual disturbances can occur during alcohol intoxication, leading to impaired vision and altered perception of the surrounding environment. These visual disturbances may include blurred vision or difficulty focusing on objects [54,55]. Euphoria can contribute to a sense of relaxation, lowered inhibitions, and increased sociability [84]. Tachycardia, irregular heartbeat, and palpitations can also occur [54,55,84]. Due to alcohol’s vasodilatory effects, which cause blood vessels to expand, resulting in heat dissipation, increased body heat and a feeling of warmth can appear. Gastrointestinal effects are nausea and vomiting (due to irritation on the lining of the stomach and gastrointestinal tract) and are frequently observed during alcohol intoxication. In extreme cases, alcohol poisoning can lead to respiratory arrest and heart failure, posing significant risks to an individual’s life [84,85].

*Benzodiazepines*. Acute benzodiazepine poisoning includes lethargy, slurred speech, ataxia, coma, and respiratory arrest, hyporeflexia, mid-pupil position, hypothermia, while chronic intoxication includes impaired motor coordination, drunkenness, feeling knee joints (due to myorelaxant action), irritability or depression, tremor of the extremities, muscle twitching, decreased appetite [51,86,87,88]. Slurred speech is a characteristic symptom that arises from the depressant effects of benzodiazepines on the central nervous system. The muscles responsible for speech become relaxed and less coordinated, leading to a noticeable impairment in articulation and clarity of speech [86,89]. Ataxia, a lack of muscle coordination, can manifest as unsteady movements, stumbling, and difficulty maintaining balance [86]. In severe cases of benzodiazepine poisoning, individuals may progress into a state of coma [87,88]. Respiratory arrest is a life-threatening complication that can occur with excessive benzodiazepine consumption. The sedative properties of benzodiazepines can depress the central nervous system, leading to suppressed respiratory function and, in severe cases, a complete cessation of breathing. Hyporeflexia, mid-pupil position, and hypothermia can also appear [88].

*Inhalants*. The use of inhalants determines euphoric excitement, palpitations, headache, dizziness, sweating, nausea, vomiting, delirium, drowsiness, tonic-clonic seizures, respiratory depression, or cardiovascular collapse but also asthenia, irritability, memory loss, restlessness, sleep disturbances, mental confusion, nausea, weight loss and loss of appetite, conjunctivitis, bronchitis, dermatitis, anemia, and leukopenia [58]. The intoxicating effects are often short-lived, leading users to repeat inhalation sessions, which can result in a cycle of abuse and increased health hazards. Prolonged and excessive inhalant use can lead to serious health consequences, including damage to the brain, liver, kidneys, lungs, and other vital organs [58,90].

2. Stimulants

*Phenethylamines* [59,61,62,91]. The use of phenethylamines can lead to a range of adverse effects that primarily affect the cardiovascular system, mental state, and overall well-being of individuals [92,93]. Some of the common adverse effects are hypertension [92], palpitations (characterized by irregular or rapid heartbeat sensations), and tachycardia, contributing to feelings of discomfort and unease [92]. Agitation and restlessness may be experienced as psychological effects of phenethylamine use [93]. Individuals may feel restless, unable to relax, and may exhibit heightened irritability or agitation. Paranoid delusions, or irrational beliefs of being persecuted or threatened, can occur during phenethylamine use. These delusions may contribute to heightened feelings of suspicion, fear, or a sense of being watched or targeted [93]. Insomnia, the inability to sleep or disturbances in sleep patterns, can be a consequence of phenethylamine use. Sleep disturbances can further exacerbate other adverse effects and impact overall well-being. Dissociation, a feeling of detachment from oneself or one’s surroundings, may be experienced during phenethylamine use [92,93]. Individuals may feel disconnected or detached from reality, contributing to a sense of unreality or detachment. Headaches, seizures, and hallucinations can also occur as a result of phenethylamine use. Panic attacks, characterized by sudden and intense feelings of fear and psychosis similar to schizophrenia, can occur as a psychological response to phenethylamine use [93].

The effects of acute consumption in the case of *amphetamine* are altered consciousness, anxiety, restlessness, headache, anorexia, confusion, bruxism, psychosis, agitation, convulsions, coma, tachycardia, hypertension, arrhythmias, tachypnea, pulmonary edema, chest pain, diaphoresis, tachypnea, and vomiting [93]. In addition, changes in behavior, such as impulsivity, have been observed [94].

The effects induced by *MDMA* were strongly correlated to the drug dose and the personality of the user, namely their ‘openness to the experience’. In this regard, studies outlined pleasant and prosocial effects in ‘open’ users, while anxious users are more likely to develop unpleasant reactions [95]. MDMA use leads to tachycardia, chest pain, bruxism, hypertension, nausea, hepatotoxicity, and cardiac arrest [96]

*Piperazines*. The use of piperazines can lead to various adverse effects, with notable concerns involving convulsions, hallucinations, and hyperthermia [78]. Hallucinations may involve visual, auditory, or tactile sensations, contributing to an altered state of perception and potential confusion. Hyperthermia is another concern associated with piperazine consumption. Piperazines have been reported to increase body temperature to dangerous levels, which can lead to severe health complications, including organ damage and even death, if not addressed promptly [78].

*Synthetic cathinone*. Agitation and restlessness are commonly observed during acute synthetic cathinone intoxication [63]. This state of heightened physical and mental activity can lead to increased impulsivity and difficulty in calming down. Panic attacks, marked by intense and overwhelming feelings of fear and distress, can also manifest during intoxication with synthetic cathinone [63,97]. These episodes can be accompanied by physical symptoms, such as rapid heartbeat, shortness of breath, chest pain, and trembling [63]. Dysphoria (a state of profound dissatisfaction, unease, and general dissatisfaction with life) may be experienced during synthetic cathinone intoxication [63,97,98]. Bizarre behavior, characterized by unusual and abnormal actions, can also be observed [63,97,98]. This behavior may include erratic movements, aggression, disorientation, or engaging in actions that are out of character. Psychosis, a severe mental condition marked by a loss of touch with reality, can occur during acute synthetic cathinone intoxication [63,97,98]. Psychotic symptoms may include hallucinations (perceiving things that are not there) and delusions (holding false beliefs). Similar to synthetic cannabinoids, the use of synthetic cathinone carries significant risks due to the potential for unpredictable and severe adverse effects.

As has already been outlined, many drug users develop bizarre behavior and even hallucinations, depending on the type of substance of abuse. Studies have shown that these changes in perceptions are likely to be correlated with opioids and various new psychoactive substances, such as cathinone and synthetic cannabinoids [99]. However, more research is needed on this topic as the survey of the literature outlined a lack of meta-analysis studies regarding ‘cathinone’, and only a few results for ‘synthetic cannabinoids’ were obtained (Figure 2).

*Khat*. Although previous systematic reviews could not associate khat use with psychiatric symptoms, it is well known that khat chewing could lead to anxiety, insomnia, mood disorders, depression, and even psychosis, and also severe psychological harms, such as suicide and homicide [64,100].

3. Particular mechanism

*Cannabis*. Acute intoxication includes euphoria and disinhibition, injected eyes, mydriasis, mild blood sugar extremities, fine tremor, impaired thinking and concentration, obsessions, delusions and hallucinations, delirium, panic, psychosis, and lack of coordination, disorganized thinking, tachycardia, and orthostatic hypotension [101,102,103,104]. The severity of these acute symptoms depends on the administration route, intravenous administration of THC leading to more severe symptoms than inhaled administration, but on the administered dose as well [102,103,104,105]. Overdose deaths without additional drug use are very rare [106].

*Synthetic cannabinoids*. Individuals who experience acute intoxication with synthetic cannabinoids may exhibit vomiting, which is a common and often immediate response to the ingestion of these substances [72,107]. Convulsions and tremors can also occur as a result of synthetic cannabinoid intoxication [107,108,109]. These involuntary muscle movements and shaking can be alarming and indicate a significant neurological impact. Behavioral changes may manifest as aggression and agitation during acute intoxication with synthetic cannabinoids [107,108,110]. This altered state of mind can lead to unpredictable and potentially dangerous actions. Slurred speech may be observed in individuals under the influence of synthetic cannabinoids [107]. The impairment of verbal communication and articulation can be indicative of the profound effects of these substances on the central nervous system [72]. High blood pressure may contribute to cardiovascular stress and potentially lead to complications if not addressed promptly [72,96,107,108,109]. Respiratory symptoms, such as wheezing and shortness of breath, can also occur [72]. These effects may be accompanied by chest discomfort and difficulty breathing, requiring medical attention to ensure proper respiratory function. In severe cases of acute intoxication, loss of consciousness may occur [72,107]. This loss of awareness and responsiveness can be indicative of a critical medical situation and necessitates immediate medical intervention. Additionally, the packaging is frequently misleading, and the use of synthetic cannabinoids carries significant risks due to the potential for unpredictable and severe adverse effects.

*Tryptamines hallucinogens*. Acute intoxication with tryptamines can result in various effects, primarily affecting perception, cognition, and physiological functions [78,111]. Individuals experiencing tryptamine intoxication may encounter visual hallucinations (which can be vivid, colorful, and highly realistic, creating an altered visual experience), de-personalization (a sense of detachment or disconnection from oneself), and alterations in sensory perception (involving heightened sensitivity to sensory stimuli, where sounds, colors, textures, and tastes may become intensified or distorted) [78,112]. In addition to perceptual effects, acute tryptamine intoxication can lead to various physiological symptoms. Anxiety (marked by feelings of unease, worry, or fear), increased blood pressure (potentially straining the cardiovascular system), headaches (contribute to the overall discomfort experienced during acute intoxication), and nausea, often accompanied by a feeling of queasiness or the urge to vomit, can also be present [112]. A meta-analysis study analyzed the post-acute psychological effects of psychedelics, and no evidence that these increase the risk for adverse effects was obtained [112]. The use of hallucinogens can trigger episodes of schizophrenia and lead to profound alteration of the consumer’s behavior. In addition, the consumer’s behavior can present physical and mental decline while presenting social implications, tachycardia, loneliness, fear, suicidal tendencies, mydriasis, hypertension, hyperthermia, tachycardia, piloerection, hyperreflexia, panic attacks, psychotic states, and flashbacks [76,77].

##### Chronic Effects

1. Depressants

Chronic intoxication with *heroin and other opioids* includes weight loss, pallor, dry skin and rash, miosis, and antisocial behavior [81].

In the case of *alcohol*, long-term consumption leads to memory disorders, tremors of the extremities, mental and physical dependence, and psychic changes that evolve into psychosis. In addition, chronic exposure to alcohol increases the risk of accidents and injuries [113] and many other chronic diseases such as heart diseases, gastrointestinal diseases, or even cancer as well [114]. The cardiovascular toxicity of alcohol has been evaluated in a meta-analysis study, and modest alcohol intake has been correlated with lower stroke incidence and mortality, while higher doses of alcohol increased cardiovascular risks, such as high blood pressure and risk of hemorrhagic stroke [115]. Still, studies have shown favorable effects on fibrinogen and high-density lipoprotein cholesterol and reduction of coronary heart disease risk in case of moderate alcohol intake [116]. In addition, the dose-response relationship has been assessed, and the outcome of a meta-analysis study was that the risk associated with alcohol consumption is sex-specific, and as expected, less consumption is better [114]. A particular situation of alcohol consumption is prenatal alcohol exposure, leading to neurocognitive deficits, facial dysmorphology, and growth deficiencies. Known as fetal alcohol spectrum disorder (FASD), the spectrum has been linked to alcohol intake, and the negative effects of higher amounts of alcohol were outlined [117].

For *benzodiazepines*, chronic intoxication includes impaired motor coordination, drunkenness, feeling knee joints (due to myorelaxant action), irritability or depression, tremors of the extremities, muscle twitching, and decreased appetite [86,87,88]. In addition, long-term use of benzodiazepines could lead to impaired cognitive functioning, psychomotor impaired functioning, as well as addiction [118].

*Inhalant* abuse can lead to addiction, with users developing a compulsion to continue using these substances despite the adverse effects on their physical and mental well-being [90].

2. Stimulants

In the case of psychoactive *phenethylamines*, long-term effects are limited [59,91].

Chronic intoxication with *amphetamines* includes psychomotor agitation, the impression of increased physical and intellectual strength, weight loss due to loss of appetite, intense thirst, and personality changes. In addition, amphetamines intake has a high risk of inducing psychoses characterized by aggressive, violent behavior and paranoid ideation. Used for therapeutical purposes, such as treatment for ADHD (attention-deficit/hyperactivity disorder), amphetamines could also lead to cardiovascular effects, such as an increase in heart rate and blood pressure [119].

Various studies showed that chronic use of *MDMA* is commonly linked with impairment of short-term and long-term memory in heavy ecstasy users, inducing clinically relevant deficits [120,121,122]. MDMA users show significantly decreased regional cerebral brain glucose metabolism (rMRGlu) in the brain, with verbal learning and recall deficits being correlated with glucose hypometabolism in the prefrontal and parietal cortex, while word recognition is correlated with medio-temporal hypometabolism [122]. Although impairment of verbal memory was identified during MDMA intoxications, a contradictory outcome of one study comparing ecstasy users to a healthy control group showed no differences between the groups [120]. The potential serotoninergic neurotoxicity of MDMA was proved, showing significant SERT reductions in the neocortical and limbic regions and damage to the serotonin axons with the longest projections from the raphe nuclei [32]. However, one study showed that the effects of long-term use of MDMA have been assessed, and no significant alterations in the brain were identified, suggesting that reductions in serotonin transporter are reversible [61].

3. Particular mechanism

*Cannabis* long-term use leads to psychotic symptoms, persistent impairment of cognitive function and memory, laryngitis, oropharyngeal cancers, periodontal disease and dental caries, chronic bronchitis, impaired endocrine function, an impaired immune system with decreased white blood cell count, tachycardia and heart disease, neuropsychiatric disorders (insomnia, disorientation, persistence of hallucinations, a passive state of the subject towards social and family life) digestive disorders (nausea, vomiting, anorexia, weight loss to cachexia) physical degradation, and mental impairment [36,104,123]. A meta-analysis demonstrated a prevalence of males with comorbid mental illness and cannabis use disorder [124], while multiple studies have assessed the connection between psychosis and cannabis use. The results outlined that cannabis users are more likely to develop psychiatric disorders such as psychosis or increased suspiciousness [71,106,125]. In addition to schizophrenia-related psychosis, it has been demonstrated that cannabis use may worsen mania symptoms in formerly bipolar-diagnosed patients [126]. One study analyzed the effect of cannabis on schizophrenic patients, and the results indicated that abstinent cannabis users were less susceptible to developing negative symptoms [127]. Associations with psychological disorders were also obtained in other studies, demonstrating higher rates of psychosis, depression, anxiety, and suicidality in the case of cannabis users [125,128]. Opposed to prior findings, a meta-analysis study evaluated the association between cannabis use and anxiety, outlining a positive relation and suggesting cannabis as a possible effective treatment of anxiety disorders [129]. Additionally, studies have analyzed the risk of developing various pathologies of testicles, such as cancer [130], or alterations in testicular functions (testosterone, gonadotropins, and semen parameters) [131], but the results were not conclusive. However, one meta-analysis study outlined the correlation between cannabis use and erectile dysfunction due to the action on the paraventricular nucleus of the hypothalamus (responsible for erectile function and sexual behavior in males) [132]. Opposed to the recreational use of cannabis, the medicinal use of cannabis (in case of chronic pain) could not be linked to serious adverse effects [133]. A particular situation of cannabis consumption is prenatal exposure, leading to decreased birth weight and the need for neonatal intensive care [134]. Recent findings outline the toxicity of cannabis on the cardiovascular system, the harmful effects on the cardiovascular system being associated with an increased risk of myocardial injury among cannabis users [135]. These findings are sustained by recent studies run on cannabis users [136].

Chronic intoxication with *synthetic cannabinoids* includes psychosis, agitation, hallucinations, delirium, hyperthermia, tachycardia, paranoia, and violent behavior [108,109,110,111], while the long-term use of synthetic cathinone leads to serious side effects, such as hallucinations, delirium, hyperthermia, and tachycardia. Symptoms such as dehydration, muscle damage, and kidney failure can lead to multiorgan failure and death [63,137]. Acting on the same receptors, the mechanism of action of synthetic cannabinoids is similar to cannabis. The survey of the literature has revealed cases of cardiovascular abnormalities in synthetic cannabinoid use, and various changes in the electrocardiogram have been outlined [138,139,140,141,142,143]. Although the literature does not provide many studies regarding the cardiovascular toxicity of cathinone, various case reports highlighted the potential toxicity of cathinone on the cardiovascular system [142]. The findings from this research are in line with the outcomes of other studies assessing the cardiotoxicity of various drugs. In this regard, previous research outlined that alcohol users are more likely to develop cardiac events than other drug users [136].

Although studies have demonstrated the potential therapeutic use of classic serotoninergic psychedelics in mood disorders and depressive symptoms [75], the adverse effects of *tryptamines* need to be taken into account. These can trigger episodes of schizophrenia and lead to profound alteration of the consumer’s behavior [76,77].

##### Withdrawal Syndrome

Most of the above-mentioned drugs possess similar profiles in the aspect of withdrawal syndrome. The main symptoms are severe agitation, anxiety, tachycardia, tremor hypertension, hyperthermia, headache, insomnia, nausea, abdominal cramps, vomiting, sweating, and disorientation.

1. Depressants

In addition, withdrawal syndrome in *heroin and other opioids* consumption includes rhinorrhea, salivary and lacrimal hypersecretion, mydriasis, myalgia, muscle spasms, excessive chills with piloerection, marked dehydration, acid-base imbalance, with possible collapse [25,51,144,145]. Moreover, studies have shown that opioids are correlated with higher levels of depressive symptoms, even in the case of therapeutical use (prescription opioid use for chronic pain). A meta-analysis study ran over 10 studies outlined an increased incidence of elevated risks of anxiety and any other mood change (such as depression) in case of prolonged opioid use as a consequence of the dysregulation of the endogenous opioid system [146].

Abstinence syndrome in the case of *alcohol* consumption includes the above-mentioned main symptoms. Hallucinations and visual illusions can also occur [55,84,147,148]. Severe shaking and seizures can also occur [25]. A meta-analysis study conducted over 50 studies outlined that younger patients tend to have more severe withdrawal. The above-mentioned symptoms of withdrawal usually appear during the first 24 h of abstinence and, if untreated, could progress to delirium tremens, the most serious manifestation of alcohol withdrawal [149].

2. Stimulants

Withdrawal syndrome in the case of *amphetamines* includes depression (which can lead to suicide), alternating with extreme agitation, fatigue, hypersomnia, hyperphagia, abdominal cramps, and gastroenteritis [25,92].

For stimulants with hallucinogenic properties such as MDMA, no typical symptoms of withdrawal were outlined [25].

Similar to other psychostimulant drugs, withdrawal syndrome for *synthetic cathinone* causes severe depression (sometimes) and headache [25]. However, more research is needed on this topic as the survey of the literature did not return any results for meta-analysis studies regarding the withdrawal effects of ‘cathinone’.

3. Particular mechanism

For *Cannabis*, signs and symptoms of abstinence (withdrawal) are tremors, nystagmus, sweating, nausea, vomiting, diarrhea, irritability, anorexia, and sleep disorders (moderate and rarely requiring medical intervention) [36,107,150,151]. Cannabis withdrawal syndrome (CWS) usually appears among regular users and includes at least three of the following: aggression/anger/irritability, anxiety/nervousness, sleep disorders, appetite disorders, restlessness, mood changes (depression), and somatic symptoms [152].

Regarding *synthetic cannabinoids*, more research is needed on this topic as the survey of the literature did not return any results for meta-analysis studies on the withdrawal effects of synthetic cannabinoids.

Withdrawal symptoms may also be experienced in the case of *inhalant* use (when an individual attempts to discontinue inhalant use), further highlighting the addictive potential of these substances.

For classical hallucinogens such as *LSD* and psilocybin, no typical symptoms of withdrawal were outlined [25].

### 2.5. Cardiovascular Effects of Drugs of Abuse

Due to the impact of drug abuse on the cardiovascular system, some information regarding the changes that may appear on a normal electrocardiogram (ECG) is to be outlined. A normal electrocardiogram should show a regular heart rate and rhythm, a typical P and T wave, the QRS complex, and a non-elevated ST segment (an early sign of myocardial infarction), nor under normal level (an early sign of heart ischemia). Sometimes, the changes that occur on the electrocardiogram can be indications of some pathologies of major clinical significance. However, due to the use of certain substances (such as amphetamines, benzodiazepines, xanthines, etc.), electrolyte imbalances, muscle manifestations, such as chills (due to hypothermia, fever or chills in withdrawal), or if patients move during the test, ECG artifacts, baseline irregularities, waveform interferences (P-wave [138,139], QRS-complex, or T-wave changes [140]), or ventricular and supraventricular arrhythmias may occur [153].

The right branch block is an electrocardiographic finding that results in the enlargement of the QRS complex and changes in the electrographic vector. It is usually benign but may also be associated with heart diseases such as myocardial infarction, heart failure, or various heart blocks and is, therefore, a predictor of mortality in certain categories of patients [154]. The left bundle branch block is an impairment of the heart’s conductivity (conduction function), usually leading to changes in ventricular mechanics resulting in cardiac remodeling. Left bundle branch block is the result of myocardial injury or hypertrophy and occurs in patients with hypertension, acute coronary synchromesh, chronic myocardial infarction, mitral and aortic valve disease, etc. When it occurs in isolation, it does not raise significant concerns in patients. When presenting with acute chest pain and syncope, its detection on ECG is of particular importance [155,156].

The aberrant ventricular complex results in a QRS complex similar to the left and right branch blocks. The presence of conduction disorders (such as hemiblocks) can prevent the diagnosis of myocardial infarction by dramatically altering the electrocardiographic manifestations, which is why, along with left ventricular hypertrophy and posterior infarction, conduction disorders should be considered a possible etiology in clinical interpretation [157,158].

Bradycardia involves a decrease in sinus heart rate below 60 bpm (beats per minute). Most patients are asymptomatic, but bradycardia can also be manifested by symptoms such as dizziness, syncope, worsening heart failure, fatigue, or fainting. In addition to the inherent etiology (ischemic heart disease, pericarditis, coronary artery disease), there are extrinsic causes, such as the administration of narcotics and cannabinoids [159].

Tachycardia, or increased heart rate, may be caused by physiological (sinus tachycardia) or pathological factors (heart diseases such as myocardial infarction, acute ischemic coronary heart disease, and heart failure). Tachycardia can also be induced by withdrawal. If the rhythm changes start in the ventricle, it is called ventricular fibrillation, and if the tachycardia originates in the atria, it is called atrial flutter [160,161].

One of the most common arrhythmias, atrial flutter, is characterized by an abnormally high heart rate with an atrial rate of over 300 beats per minute and a ventricular rate that can be constant or variable. This manifestation causes palpitations, fatigue, syncope, and embolic phenomena. More frequently in men than in women, atrial flutter may be associated with risk factors such as old age, hypertension, and a history of alcohol abuse [162].

Although most of the above-mentioned substances of abuse lead to cardiac manifestations, only two meta-analyses studies linked a specific cardiovascular pathology with drug use. In both studies, alcohol consumption has been linked to a high risk of coronary heart disease [116,117]. In addition, recent findings outline the toxicity of cannabis on the cardiovascular system [135] and the toxicity of synthetic cannabinoids (leading to cardiac arrest, arrhythmias, but also infarction, hypertension, tachycardia, or bradycardia) [141].

## 3. Limitations

A few limitations of this research are to be outlined. First of all, the present review was not intended to be a systematic one. Although the database was carefully surveyed, it is possible that not all studies in the field were available or accessible on PubMed, leading to the necessity of more research on other databases as well. Regarding the risk of bias in the included studies, although most of the analyzed studies included possible bias in their research, there is still a risk that the studies included in the systematic review may have methodological flaws or biases that could influence the overall results. Although we included, in our research, studies with negative or non-significant results as well, there is a possibility that the results could have been overestimated due to publication bias (a tendency for the publication of positive-results studies).

## 4. Materials and Methods

**Figure 4:**
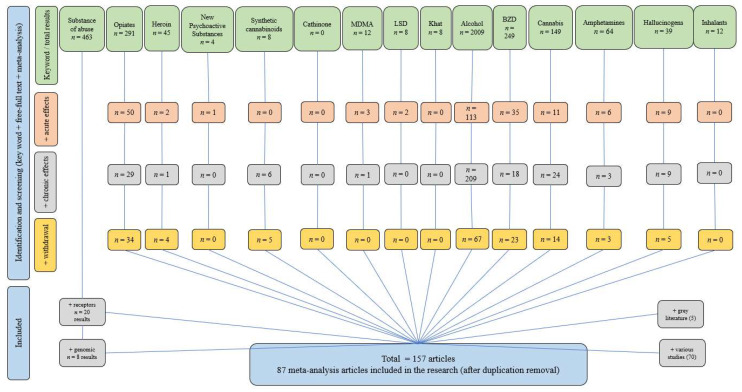
Flowchart of the literature search and selection criteria for articles on substance use disorders.

A literature survey was performed by searching the PubMed database, with no time limit. The selection of publications was performed by using keywords such as alcohol, amphetamines, benzodiazepines, cannabis, cathinone, hallucinogens, inhalants, new psychoactive substances, opioids, heroin, khat, MDMA, LSD, and synthetic cannabinoids combined with either ‘acute’, ‘chronic’, and ‘withdrawal’. The main criteria of article choice were complexity in describing the mechanism of action of the above-mentioned types of drugs, information regarding acute and chronic effects after drug intake, and information regarding the onset of abstinence syndrome. Both review and original articles were taken into consideration. In addition, the database was surveyed using the wording ‘substance of abuse’. In all cases, ‘meta-analysis’ and ‘free full-text’ were ticked, but articles without filters were also taken into account. The exclusion criteria were: *irrelevant topics* (were excluded the articles that did not specifically address the mechanism of action, acute and chronic effects, or withdrawal symptoms of the listed types of drugs), *duplicate publications* (avoid redundancy and ensure the inclusion of unique information), *insufficient information* (were excluded the articles that lacked comprehensive information or did not provide substantial details about the mechanism of action, acute and chronic effects, or withdrawal symptoms of the drugs in question), *inaccessible articles* (articles that were not available as free full-text were excluded), and *lack of relevance to substance abuse* (articles that discussed substances not related to abuse or addiction were excluded). The research approached a total of 157 articles, of which 87 meta-analyses. The flowchart of the literature search is synthesized in Figure 4.

## 5. Conclusions

The presented toxicological data clearly provides a more complete picture of the current state of knowledge on this topic. In addition, using a rigorous and transparent methodology for selecting, appraising, and synthesizing studies, this paper reduces the risk of errors and inconsistencies in the review process and ensures that the results are as accurate and reliable as possible. Furthermore, the outcome of this study can provide evidence to help healthcare providers differentiate various groups of drug users. Synthetizing the mechanism of action for the main groups of substances of abuse, the outcome of the study provides, therefore, a future perspective on the drug abuse approach.

## Figures and Tables

**Table 1:** Mechanism of action of various substances of abuse on receptors/systems.

Receptor/System	Abuse Substance (Types of Receptors)
Opioidergic	Heroin (µ, κ, δ), alcohol (µ), cocaine (µ, κ, Σ);
GABAminergic	Alcohol (GABA_A_, GABA_C_), benzodiazepines (GABA_2α_, GABA_1α_);
Serotoninergic	Synthetic Cathinones (serotonin transporters 5-HT), LSD, amphetamines (5-HT_1A_, 5-HT_2A_, 5-HT_2C_), alcohol (5-HT_3_);
Dopaminergic	Synthetic Cathinones (dopamine transporters DAT), LSD (D_2_, D_3_), amphetamines, alcohol;
Adrenergic	Synthetic Cathinones (norepinephrine transporters NET), LSD (α_2_), amphetamines, cocaine;
Cannabimimetic	Synthetic cannabinoids (CB_1_, CB_2_), THC (CB_1_, CB_2_), alcohol.

## Data Availability

The data presented in this study are available on request from the corresponding author.
